# Sex differences in clinical outcomes after transcatheter aortic valve implantation: a real-world cohort

**DOI:** 10.3389/fcvm.2026.1941651

**Published:** 2026-10-20

**Authors:** Miriam Estévez Táboas, Julio Echarte-Morales, Ernesto José Ruiz Golcher, Carlos Eduardo Sotomayor-Casillas, André González García, David Neuhalfen, Carla Iglesias-Otero, Elena García-Campos Losada, Nazaret Vázquez-Vázquez, David González Fernández, Hugo Prado Prado, Carmen Cuiñas Fariña, Andrés Antelo-Abeijón, Pablo Juan-Salvadores, Rodrigo Estevez-Loureiro, Jose A. Baz Alonso, Andres Iñiguez-Romo, Victor Alfonso Jímenez-Díaz

**Affiliations:** 1 Cardiology, Hospital Alvaro Cunqueiro, Vigo, Spain; 2 Cardiovascular Research Group, Galicia Sur Health Research Institute (IIS Galicia Sur), Vigo, Spain; 3 Interventional Cardiology Clinic, University Hospital Alvaro Cunqueiro, Vigo, Spain; 4 Servicio de Cardiología, Hospital Rafael Ángel Calderón Guardia, San José, Costa Rica; 5 Cardiología. Hospital Civil de Guadalajara “Fray Antonio Alcalde”, Universidad de Guadalajara, Centro Universitario de Ciencias de la Salud, Guadalajara, Jalisco, Mexico

**Keywords:** aortic stenosis, bleeding, mortality, permanent pacemaker implantation, sex differences, transcatheter aortic valve implantation, vascular complications

## Abstract

**Background:**

Severe aortic stenosis is one of the most prevalent valvular heart diseases in the ageing population, and transcatheter aortic valve implantation (TAVI) has become an effective and less invasive therapeutic alternative. Anatomical, pathophysiological, and clinical differences between women and men may influence outcomes following the procedure. This study aimed to evaluate the association between sex and mortality and procedure-related complications after TAVI in a real-world cohort from the Vigo Health Area.

**Methods:**

We conducted a retrospective, single-center observational study including 1,065 patients with severe aortic stenosis who underwent transfemoral TAVI between 2010 and 2023. The primary endpoint was all-cause mortality at 1 year. Secondary endpoints included bleeding, vascular complications, and new permanent pacemaker implantation. Time-to-event analyses were censored at 365 days; multivariable models used complete-case analysis.

**Results:**

Women were older and had greater frailty and higher surgical risk scores than men. In-hospital and 1-year mortality were similar between sexes. Date-based 30-day mortality was 2.4% in men and 3.7% in women (*p* = 0.225), and female sex was not independently associated with 30-day mortality after adjustment for age and STS score (adjusted OR 1.79, 95% CI 0.80–3.98; *p* = 0.154). Female sex was not independently associated with 1-year mortality (adjusted HR 1.07, 95% CI 0.69–1.66; *p* = 0.772). Women had more bleeding in unadjusted analyses, but female sex was not significantly associated with any bleeding after adjustment (adjusted OR 1.61, 95% CI 0.92–2.81; *p* = 0.093). Male sex remained associated with higher odds of new permanent pacemaker implantation (adjusted OR 1.49, 95% CI 1.03–2.17; *p* = 0.036).

**Conclusions:**

Sex was not independently associated with short- or 1-year mortality after TAVI. Women had a higher unadjusted incidence of bleeding, whereas men had a higher incidence of new permanent pacemaker implantation that persisted after adjustment. These findings describe sex-related differences in procedural risk profiles but should be interpreted in the context of the observational design and the long study period.

## 1 Introduction

Aortic stenosis (AS) is the most common valvular heart disease requiring interventional treatment in Europe and North America. Progressive valve calcification and fibrosis lead to left ventricular pressure overload, remodeling, and eventually heart failure (1, 2). Disease progression accelerates with increasing severity (3). Although patients may remain asymptomatic for years, symptom onset is associated with a marked deterioration in prognosis, making aortic valve replacement the only treatment that improves survival (1, 4, 5).

The prevalence of AS increases markedly with age, affecting approximately 0.2% of adults aged 50–59 years and nearly 10% of those aged 80–89 years (6). Population ageing is therefore expected to substantially increase the need for valve replacement (7).

Since its introduction in 2002, Transcatheter aortic valve implantation (TAVI) has transformed the management of severe symptomatic AS (8). Evidence supporting its use across the surgical-risk spectrum has expanded its indications (9–11), while advances in technology, procedural planning, and implantation techniques have improved safety and clinical outcomes (12).

Despite these advances, important sex-related differences persist among patients with AS. Women present distinct anatomical and pathophysiological characteristics, including smaller aortic root dimensions (13, 14), lower degrees of aortic valve calcification for the same hemodynamic severity (15), and a predominance of concentric left ventricular remodeling characterized by greater ventricular stiffness and diastolic dysfunction, whereas men more frequently develop eccentric remodeling with greater ventricular dilatation (16). Sex-related differences have also been described in myocardial fibrosis patterns, with women exhibiting more diffuse fibrosis and men more focal fibrosis, which may influence the reversibility of myocardial damage after valve replacement (17). These structural differences contribute to distinct clinical phenotypes, with women more frequently presenting heart failure with preserved ejection fraction and paradoxical low-flow low-gradient aortic stenosis (18).

Sex-related physiological differences also influence the clinical presentation of aortic stenosis. Women more commonly present dyspnea, dizziness and reduced exercise tolerance, which have been attributed to a higher prevalence of coronary microvascular dysfunction, smaller left ventricular cavity size and mass associated with diastolic dysfunction, as well as concomitant mitral or tricuspid valve disease (19, 20). In addition, women are more likely to develop symptoms during follow-up despite having a similar degree of AS severity at baseline (21). Conversely, men more frequently present with exertional angina, probably reflecting the higher prevalence of concomitant coronary artery disease (22). Although symptoms such as chest pain, dyspnea, dizziness and syncope occur in both sexes, they are generally more pronounced in women. This greater symptom burden is partly explained by their older age at presentation, higher frailty, lower body mass index, poorer baseline New York Heart Association (NYHA) functional class and higher surgical risk, as reflected by higher Society of Thoracic Surgeons (STS) risk scores (20, 23).

Evidence also suggests that women with AS are frequently diagnosed at more advanced stages of the disease and present a higher-risk clinical profile, characterized by older age, greater symptom burden, increased frailty, impaired renal function and a higher prevalence of symptomatic heart failure (24). Beyond biological differences, important sex-related disparities exist throughout the diagnostic and therapeutic pathway. A nationwide Spanish study showed that women were less likely to be admitted to cardiology or cardiac surgery departments and were more frequently managed in internal medicine, despite having a higher prevalence of severe AS and congestive heart failure than men. Women were also less likely to undergo diagnostic investigations, including echocardiography and coronary angiography, and were referred less frequently for surgical aortic valve replacement, although referral rates for TAVI were comparable between sexes (25).

Several factors may contribute to these disparities, including prolonged conservative management, advanced age, frailty, underestimation of disease severity, reluctance to undergo invasive diagnostic procedures and less favorable preoperative clinical characteristics. Although TAVI is currently performed with similar frequency in women and men, women continue to experience longer waiting times before intervention, which have been associated with higher 30-day mortality and increased hospitalizations for heart failure (23).

Collectively, these findings suggest that sex-related bias may contribute to delays in the diagnosis and treatment of women with AS, as healthcare professionals may underestimate both symptom severity and disease severity in female patients (19). These observations underscore the importance of incorporating a sex-specific perspective throughout the diagnostic pathway to facilitate earlier recognition, timely referral and equitable access to appropriate treatment.

Therefore, further investigation of sex-related differences among patients undergoing TAVI is warranted. A better understanding of these differences may improve patient selection, support individualized treatment strategies, and contribute to reducing sex-related disparities in the management of aortic stenosis.

## 2 Materials and methods

### 2.1 Study design and population

We performed a retrospective, single-center observational cohort study including consecutive patients with symptomatic severe native aortic stenosis who underwent transfemoral TAVI between 2010 and 2023 at the Álvaro Cunqueiro University Hospital Complex in Vigo, Spain. Patients were evaluated by a multidisciplinary Heart Team, which determined the indication for TAVI according to guidelines and institutional practice applicable at the time of the procedure.

The study evaluated the association between biological sex and mortality and procedure-related outcomes. Data were obtained from the institutional TAVI registry and electronic medical records. The retrospective design enabled analysis of a long real-world procedural period, but no causal inference was intended.

Inclusion criteria

1. Symptomatic severe native aortic stenosis.
2. Procedure performed on native aortic valve.
3. Availability of sufficient information to confirm TAVI eligibility and ascertain the principal study outcomes.

Exclusion criteria

1. TAVI performed for pure aortic regurgitation as the primary indication.
2. Non-transfemoral access.

Patients were not excluded solely because an individual laboratory, echocardiographic, or follow-up covariate was missing. Variable-specific denominators are reported in the tables. Missing covariate data were not imputed; multivariable analyses used complete-case analysis.

### 2.2 Data collection

Clinical, echocardiographic and procedural data were extracted from the institutional TAVI registry and complemented through review of the electronic medical record.

Baseline demographic, clinical, laboratory, echocardiographic, procedural, and follow-up data were collected retrospectively from the institutional TAVI registry and the electronic medical record. Baseline demographic variables included age and sex. Clinical variables comprised cardiovascular risk factors, comorbidities, frailty, New York Heart Association (NYHA) functional class, previous cardiovascular interventions, and surgical risk assessed using the EuroSCORE II and Society of Thoracic Surgeons (STS) score. Baseline echocardiographic assessment included left ventricular ejection fraction, transvalvular gradients, aortic valve area, valve morphology, pulmonary artery systolic pressure, and concomitant valvular disease. Procedural variables included vascular access, prosthesis type and size, device category, contrast volume, and procedural success. Follow-up data included survival status, hospital readmissions, NYHA functional class, and echocardiographic assessment at scheduled follow-up visits.

### 2.3 Outcomes

The primary endpoint was all-cause mortality at 1 year after TAVI. Mortality was additionally assessed during the index hospitalization and at 30 days; 30-day mortality was reconstructed from the TAVI and death dates. Secondary endpoints included bleeding complications, vascular complications, and new permanent pacemaker implantation. The institutional registry was created over a long period and predominantly used Valve Academic Research Consortium-2 (VARC-2) categories for procedural events. Bleeding was recorded as none, minor, major, or life-threatening/disabling; “any bleeding” comprised any non-zero category, and “major bleeding” comprised major or life-threatening/disabling bleeding. Vascular complications and cerebrovascular events were analyzed using the VARC-2 categories available in the registry. Events were verified against the electronic medical record when needed but were not adjudicated by an independent clinical-events committee. New permanent pacemaker implantation was evaluated only among patients without a pre-existing pacemaker and with available post-TAVI pacemaker status.

### 2.4 Statistical analysis

Continuous variables are presented as mean ± standard deviation or median [interquartile range], according to distribution. Categorical variables are expressed as absolute frequencies and percentages calculated using patients with available data as the denominator.

Comparisons between women and men were performed using the Student's *t*-test or Mann–Whitney *U* test for continuous variables and the chi-square test or Fisher's exact test for categorical variables, as appropriate.

Time-to-event analyses were administratively censored at 365 days. Patients without an event were censored at the date of last available follow-up or at 365 days, whichever occurred first. Vital status was ascertained from the institutional TAVI registry and electronic medical records. Cumulative all-cause mortality was estimated using the Kaplan–Meier method, and differences between sexes were assessed using the log-rank test. Independent predictors of 1-year mortality were assessed using multivariable Cox proportional hazards regression including age, sex, frailty, hemoglobin, eGFR, atrial fibrillation, STS score, LVEF, and COPD. Missing covariate data were handled using complete-case analysis without imputation. The proportional hazards assumption was assessed using Schoenfeld residuals. A parsimonious logistic regression model for 30-day mortality included sex, age, and STS score. Multivariable logistic regression analyses were also performed for any bleeding and new permanent pacemaker implantation. The bleeding model included sex, age, body mass index, baseline hemoglobin, and eGFR. The pacemaker model included sex, age, prosthesis category, and prosthesis size; patients with a pre-existing pacemaker were excluded. Sensitivity analyses additionally included procedure year as a continuous covariate. A sex-by-prosthesis-category interaction term was tested for new pacemaker implantation. Adjusted hazard ratios (HRs) or odds ratios (ORs) with 95% confidence intervals are reported. A two-sided *p* value < 0.05 was considered statistically significant. Analyses were performed using IBM SPSS Statistics version 29.0 and Stata version 16.0.

## 3 Results

A total of 1,065 consecutive patients with severe symptomatic aortic stenosis who underwent transfemoral TAVI between 2010 and 2023 were included in the study. Of these, 519 (48.7%) were women and 546 (51.3%) were men.

### 3.1 Baseline clinical characteristics

Women were slightly older than men (84 [81–87] vs. 83 [78–86] years; *p* < 0.001) and had a higher prevalence of hypertension (86.3% vs. 81.3%; *p* = 0.022). They also exhibited higher surgical risk scores, with significantly higher EuroSCORE II (4.4 [2.5–7.2] vs. 3.4 [1.9–6.4]; *p* < 0.001) and STS scores (5.9 [3.8–9.3] vs. 4.6 [3.0–6.6]; *p* < 0.001). Likewise, frailty was more frequently observed in women than in men (28.3% vs. 23.1%; *p* = 0.050).

In contrast, men had a higher prevalence of diabetes mellitus (38.5% vs. 31.8%; *p* = 0.023) and chronic obstructive pulmonary disease (17.8% vs. 10.8%; *p* = 0.001). Men also had higher baseline serum creatinine levels (1.1 [0.9–1.4] vs. 0.9 [0.8–1.2] mg/dL; *p* < 0.001), whereas women had lower estimated glomerular filtration rates (53 [38–69.2] vs. 57 [42–72.3] mL/min/1.73 m^2^; *p* = 0.047).

Regarding cardiovascular history, men were more likely to have undergone previous coronary artery bypass grafting (8.4% vs. 5.2%; *p* = 0.039), whereas no significant sex differences were observed for the remaining baseline variables. Baseline characteristics of the study population are summarized in Table 1.

**Table 1:** Baseline characteristics.

Clinical characteristics	All patients	Men	Women	*p*
	(*N* = 1,065)	(*n* = 546)	(*n* = 519)	
Demographic characteristics				
Age, years (*n* = 1,065)	83 (79–87)	83 (78–86)	84 (81–87)	<0.001
Body mass index, kg/m^2^ (*n* = 1,057)	27.8 (25.1–31.1)	27.9 (25.5–30.9)	27.6 (24.6–31.2)	0.299
Clinical record				
Frailty (*n* = 1,063)	273 (25.7)	126 (23.1)	147 (28.4)	0.050
Hypertension (*n* = 1,064)	892 (83.8)	444 (81.3)	448 (86.5)	0.022
Diabetes mellitus (*n* = 1,065)	375 (35.2)	210 (38.5)	165 (31.8)	0.023
COPD (*n* = 1,064)	153 (14.4)	97 (17.8)	56 (10.8)	0.001
Hemoglobin (*n* = 1,061)	11.9 ± 1.7	12.2 ± 1.81	11.7 ± 1.5	<0.001
Creatinine (*n* = 1,063)	1 (0.8–1.3)	1.1 (0.9–1.4)	0.9 (0.8–1.2)	<0.001
GFR (*n* = 904)	55.2 (40–71)	57 (42–72.3)	53 (38–69.2)	0.047
NYHA (*n* = 1,060)	417 (39.3)	197 (36.2)	220 (42.6)	0.089
1	22 (2.1)	9 (1.7)	13 (2.5)	
2	621 (58.6)	338 (62.1)	283 (54.8)	
3	395 (37.3)	188 (34.6)	207 (40.1)	
4	22 (2.1)	9 (1.7)	13 (2.5)	
Previous AMI (*n* = 1,060)	104 (9.8)	57 (10.5)	47 (9.1)	0.442
Previous PCI (*n* = 1,060)	211 (19.9)	112 (20.6)	99 (19.1)	0.547
Previous coronary artery bypass surgery (*n* = 1,061)	73 (6.9)	46 (8.4)	27 (5.2)	0.039
Previous valve surgery (*n* = 1,062)	60 (5.6)	27 (5.0)	33 (6.4)	0.313
Atrial fibrillation (*n* = 1,058)	348 (32.9)	180 (33.1)	168 (32.6)	0.855
Pacemaker (*n* = 1,061)	103 (9.7)	51 (9.4)	52 (10.1)	0.707
EuroScore II, % (*n* = 1,062)	3.8 (2.2–6.8)	3.4 (1.9–6.4)	4.4 (2.5–7.2)	<0.001
STS-Score, % (*n* = 882)	5.2 (3.4–8.0)	4.6 (3.0–6.6)	5.9 (3.8–9.3)	<0.001

BMI, body mass index; COPD, chronic obstructive pulmonary disease; eGFR, estimated glomerular filtration rate; NYHA, New York Heart Association functional class; MI, myocardial infarction; PCI, percutaneous coronary intervention; EuroSCORE II, European System for Cardiac Operative Risk Evaluation II; STS, Society of Thoracic Surgeons risk score.

Data are presented as *n* (%), mean ± standard deviation (SD), or median [interquartile range (IQR)].

### 3.2 Baseline echocardiographic characteristics

Baseline echocardiographic findings are presented in Table 2.

**Table 2:** Echocardiographic characteristics.

Variables	All patients	Men	Women	*p*
	(*N* = 1,065)	(*n* = 546)	(*n* = 519)	
Echocardiographic characteristics				
LVEF, % (*n* = 1,062)	55 (50–60)	55 (48–60)	60 (55–60)	<0.001
Aortic peak gradient, mmHg (*n* = 759)	76 (66.6–88.2)	76 (66–87.5)	76 (67–90)	0.497
Aortic mean gradient, mmHg (*n* = 1,034)	45 (40–53)	44 (37.7–51.6)	45.3 (40.9–55)	<0.001
Aortic valve area, cm^2^ (*n* = 613)	0.7 (0.6–0.8)	0.7 (0.6–0.9)	0.7 (0.6–0.8)	0.010
Etiology MR (*n* = 598)	371 (62.0)	174 (58.6)	197 (65.4)	0.084
Grade MR (*n* = 1,039)	74 (7.1)	38 (7.1)	36 (7.2)	0.966
Moderate—Severe	67 (6.4)	34 (6.3)	33 (6.6)	
Severe	7 (0.7)	4 (0.7)	3 (0.6)	
TR grade (*n* = 1,032)	65 (6.3)	27 (5.1)	38 (7.6)	0.102
Moderate—Severe	44 (4.3)	19 (3.6)	25 (5.0)	
Severe	21 (2.0)	8 (1.5)	13 (2.6)	
AR grade (*n* = 1,038)	42 (4.0)	24 (4.5)	18 (3.6)	0.436
Moderate—Severe	32 (3.1)	18 (3.4)	14 (2.8)	
Severe	10 (1.0)	6 (1.1)	4 (0.8)	
Bicuspid aortic valve (*n* = 1,048)	16 (1.5)	9 (1.7)	7 (1.4)	0.686
SPAP, mmHg (*n* = 502)	40 (31–50)	40 (31–49)	40 (31.8–50)	0.377

LVEF, left ventricular ejection fraction; MR, mitral regurgitation; TR, tricuspid regurgitation; AR, aortic regurgitation; SPAP, systolic pulmonary artery pressure.

Data are presented as *n* (%), mean ± standard deviation (SD), or median [interquartile range (IQR)].

Women had a significantly higher left ventricular ejection fraction than men (60 [55–60] vs. 55 [48–60]; *p* < 0.001), as well as higher mean transaortic gradients (45.3 [40.9–55] vs. 44 [37.7–51.6]; *p* < 0.001). In contrast, men showed a slightly larger aortic valve area (*p* = 0.010).

### 3.3 Procedural characteristics

The prosthesis size implanted was significantly smaller in women compared with men (26 [26–29] mm vs. 29 [27–31]; *p* < 0.001). Procedural success was high in both groups, although slightly higher in men (98.9% vs. 97.3%; *p* = 0.044). No significant differences were observed in device type or expansion mechanism between sexes. In addition, men received a higher contrast volume during the procedure (216.5 [180–264.3] vs. 200 [160–249]; *p* = 0.001). Other procedural variables are detailed in Table 3.

**Table 3:** Procedure characteristics.

Variables	All patients	Men	Women	*p*
	(*N* = 1,065)	(*n* = 546)	(*n* = 519)	
Procedure characteristics				
Device size (*n* = 1,056)	28 (26–29)	29 (27–31)	26 (26–29)	<0.001
Success of the procedure (*n* = 1,063)	1,045 (98.3)	540 (99.1)	505 (97.5)	0.044
Device				
Sapien (*n* = 1,065)	185 (17.4)	100 (18.3)	85 (16.4)	0.404
Evolut (*n* = 1,065)	515 (48.4)	272 (49.8)	243 (46.8)	0.328
Navitor (*n* = 1,065)	161 (15.1)	72 (13.2)	89 (17.1)	0.071
MyVal (*n* = 1,065)	2 (0.2)	2 (0.2)	0	0.500
Allegra (*n* = 1,065)	145 (13.6)	68 (12.5)	77 (14.8)	0.257
Other devices: ES, HYDRA, Direct Flow, Accurate, CVP (*n* = 1,065)	56 (5.3)	31 (5.7)	25 (4.8)	0.529
Types of prostheses (*n* = 1,064)				0.285
Balloon-expandable	190 (17.9)	104 (19.1)	86 (16.6)	
Self-expanding	874 (82.1)	441 (80.9)	433 (83.4)	
Contrast (*n* = 589)	209 (170–260)	216.5 (180–264.3)	200 (160–249)	0.001

Data are presented as *n* (%), mean ± standard deviation (SD), or median [interquartile range (IQR)].

### 3.4 Mortality

No significant differences in mortality were observed between women and men during hospital admission or at 30 days after TAVI. In-hospital mortality was 2.0% in men and 3.5% in women (*p* = 0.144). In the date-based survival population, 30-day mortality was 13/543 (2.4%) in men and 19/518 (3.7%) in women (*p* = 0.225). In a parsimonious multivariable logistic model including sex, age, and STS score, female sex was not independently associated with 30-day mortality (adjusted OR 1.79, 95% CI 0.80–3.98; *p* = 0.154).

The time-to-event analysis included 1,061 of the 1,065 patients in the overall cohort (543 men and 518 women). Four patients (3 men and 1 woman) with documented death but without an ascertainable date of death were excluded. One-year vital status was ascertained in 1,005 patients (94.7%), while 56 patients (5.3%) were censored before 365 days. A total of 121 deaths occurred within 1 year. The crude proportion of 1-year deaths was 11.6% (63/543) in men and 11.2% (58/518) in women. Kaplan–Meier estimated cumulative all-cause mortality at 1 year was 11.9% (95% CI 9.4–14.9) in men and 11.4% (95% CI 9.0–14.6) in women, with no significant difference between groups (log-rank *p* = 0.879) (Figure 1). The slightly higher Kaplan–Meier estimates compared with the crude proportions reflect right censoring before 1 year.

**Figure 1:**
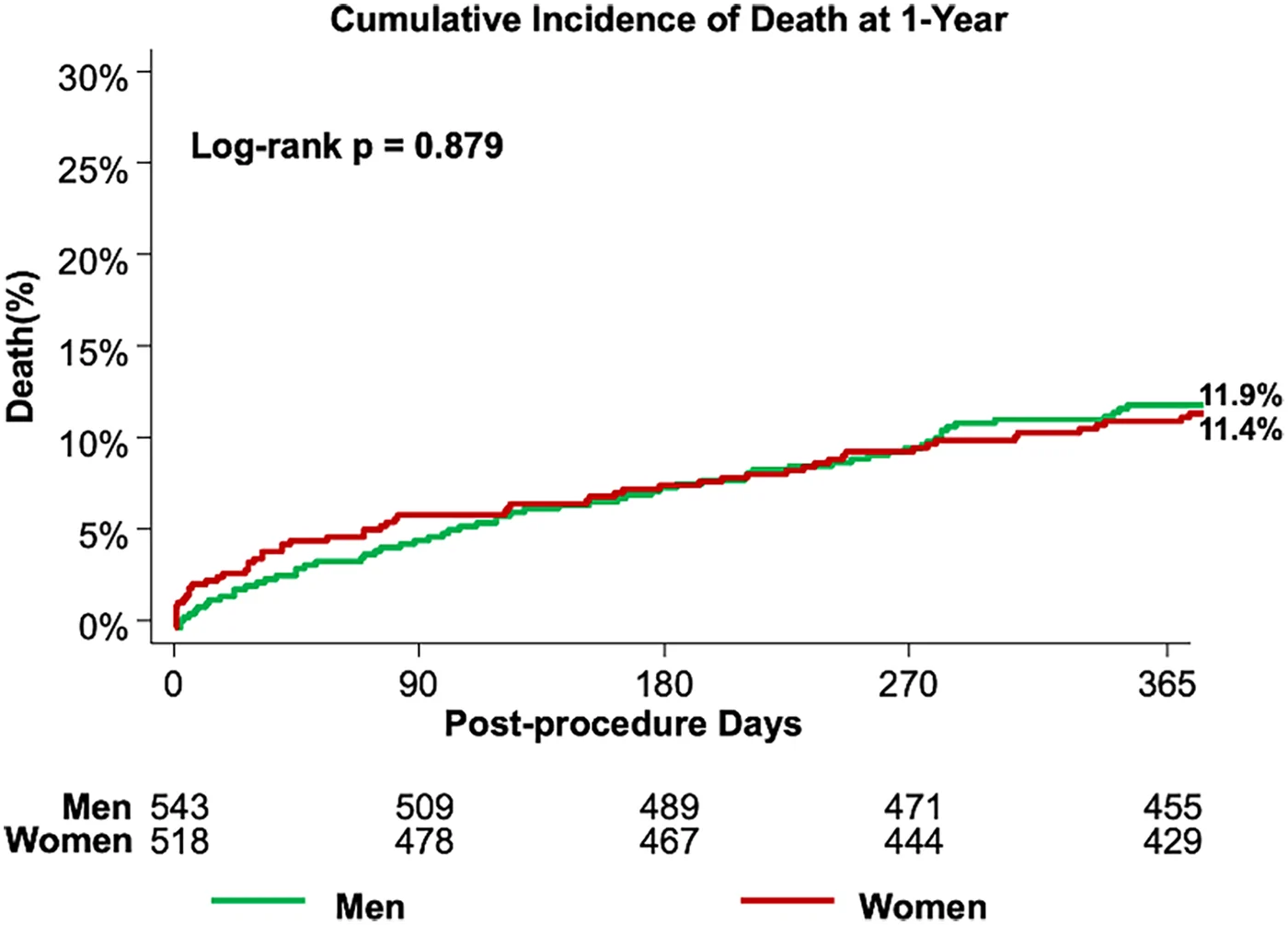
Kaplan–meier curves for all-cause mortality during the first year of follow-up after TAVI by sex.

### 3.5 Procedural complications

The overall incidence of procedure-related complications was low and similar between men and women for most of the evaluated outcomes. Detailed results are presented in Table 4.

**Table 4:** Clinical outcomes.

Variables	All patients	Men	Women	*p*
	(*N* = 1,065)	(*n* = 546)	(*n* = 519)	
Days of hospitalization (*n* = 1,058)	6.0 (5.0–9.0)	6.0 (5.0–8.5)	6.0 (5.0–9.0)	0.980
Clinical outcomes				
In-hospital mortality (*n* = 1,064)	29 (2.7)	11 (2.0)	18 (3.5)	0.144
30-day mortality (*n* = 1,061)	32 (3.0)	13 (2.4)	19 (3.7)	0.225
1-year mortality, Kaplan Meier estimate, % (95% CI) (*n* = 1,061)^a^	11.6 (9.8–13.7)	11.9 (9.4–14.9)	11.4 (9.0–14.6)	0.879
TAVI-related complications				
Coronary occlusion (*n* = 1,060)	3 (0.3)	0 (0.0)	3 (0.6)	0.114
Tamponade (*n* = 1,062)	12 (1.1)	3 (0.5)	9 (1.7)	0.067
Annular rupture (*n* = 1,055)	5 (0.5)	2 (0.4)	3 (0.6)	0.679
Mitral regurgitation (*n* = 736)	24 (3.3)	15 (3.8)	9 (2.6)	0.349
Moderate—Severe	19 (2.6)	12 (3.1)	7 (2.0)	
Severe	5 (0.7)	3 (0.8)	2 (0.6)	
Aortic regurgitation (*n* = 882)	14 (1.6)	7 (1.5)	7 (1.6)	0.911
Moderate—Severe	8 (0.9)	4 (0.9)	4 (0.9)	
Severe	6 (0.7)	3 (0.7)	3 (0.7)	
Aortic mean gradient, mmHg (*n* = 480)	8 (5.5–12)	8.2 (6.0–12.8)	8 (5.0–12.0)	0.147
CVA/TIA (*n* = 1,052)				0.776
TIA	23 (2.2)	11 (2.0)	12 (2.4)	0.713
CVA	16 (1.5)	10 (1.8)	6 (1.2)	0.380
AMI (*n* = 1,049)	4 (0.4)	1 (0.2)	3 (0.6)	0.358
AKI (*n* = 1,045)				0.746
Stage 1	84 (8.0)	44 (8.2)	40 (7.9)	
Stage 2	18 (1.7)	11 (2.0)	7 (1.4)	
Stage 3	10 (1.0)	4 (0.7)	6 (1.2)	
Vascular complications (*n* = 1,051)	54 (5.1)	21 (3.9)	33 (6.5)	0.059
Bleeding (*n* = 1,063; VARC-2 registry categories)				
Any type	73 (6.9)	26 (4.8)	47 (9.1)	0.006
Major or life-threatening/disabling	25 (2.4)	7 (1.3)	18 (3.5)	0.018
Life-threatening/disabling	3 (0.3)	1 (0.2)	2 (0.4)	0.615
New permanent pacemaker (*n* = 932; men *n* = 483, women *n* = 449^b^)	210 (22.5)	130 (26.9)	80 (17.8)	0.001
Cardiac readmission (*n* = 964; men *n* = 490, women *n* = 474)	196 (20.3)	95 (19.4)	101 (21.3)	0.459
12-month NYHA (*n* = 496; men *n* = 250, women *n* = 246)				0.218
1	180 (36.3)	100 (40.0)	80 (32.5)	
2	269 (54.2)	127 (50.8)	142 (57.7)	
3	47 (9.5)	23 (9.2)	24 (9.8)	

CVA, cerebrovascular accident; TIA, transient ischemic attack; MI, myocardial infarction; AKI, acute kidney injury; NYHA, New York Heart Association functional class. Procedural events reflect the VARC-2 categories recorded in the institutional registry.

a One-year cumulative all-cause mortality is presented as the Kaplan–Meier estimate with 95% confidence interval; *p* value from log-rank test. The Kaplan–Meier analysis included 1,061 patients (543 men and 518 women).

b Patients with a pre-existing permanent pacemaker or unavailable post-TAVI pacemaker status were excluded. Percentages use available-case denominators.

Data are presented as *n* (%), mean ± standard deviation (SD), or median [interquartile range (IQR)].

Regarding mechanical complications, coronary occlusion was an infrequent event (0.3%), observed only in women (0.6% vs. 0%; *p* = 0.114). Similarly, the incidence of cardiac tamponade was numerically higher in women (1.7% vs. 0.5%; *p* = 0.067), although without reaching statistical significance. Annular rupture was rare (0.5%) and showed no significant differences between groups (*p* = 0.679).

Concerning valve-related outcomes, no significant differences were observed in postprocedural aortic regurgitation, mean aortic gradient at discharge, or moderate/severe mitral regurgitation at 30 days. The peak-gradient variable was not retained in the revised analysis because review of the registry demonstrated non-uniform historical coding across the long study period.

With respect to cerebrovascular events, no significant differences were observed in the incidence of stroke (1.8% vs. 1.2%; *p* = 0.380) or transient ischemic attack (2.0% vs. 2.3%; *p* = 0.713). Similarly, the incidence of periprocedural myocardial infarction was low (0.4%) and comparable between groups (*p* = 0.358).

Regarding renal complications, the incidence of acute kidney injury was similar between men and women (*p* = 0.746), with no relevant differences across severity stages.

Vascular complications showed a non-significant unadjusted trend toward a higher incidence in women (6.5% vs. 3.9%; *p* = 0.059). Any bleeding was more frequent in women in the unadjusted analysis (9.1% vs. 4.8%; *p* = 0.006), as was major or life-threatening/disabling bleeding (3.5% vs. 1.3%; *p* = 0.018), whereas life-threatening/disabling bleeding alone was rare (0.4% vs. 0.2%; *p* = 0.615). After adjustment for age, body mass index, baseline hemoglobin, and eGFR, female sex was not significantly associated with any bleeding (adjusted OR 1.61, 95% CI 0.92–2.81; *p* = 0.093). Among patients without a pre-existing permanent pacemaker and with available post-TAVI pacemaker status (*n* = 932), new permanent pacemaker implantation was more frequent in men than in women (26.9% vs. 17.8%; *p* = 0.001). After adjustment for age, prosthesis category, and prosthesis size, male sex remained associated with higher odds of new pacemaker implantation (adjusted OR 1.49, 95% CI 1.03–2.17; *p* = 0.036). The sex-by-prosthesis-category interaction was not significant (*p* = 0.953), providing no evidence that the association between sex and pacemaker implantation differed between self-expanding and balloon-expandable prostheses. Sensitivity models including procedure year produced materially unchanged sex estimates for bleeding and pacemaker implantation.

### 3.6 Last follow-up

At the latest available follow-up (Table 5), NYHA functional class was available in 835 patients (422 men and 413 women), with no significant sex difference (*p* = 0.401). Among patients with available data, NYHA class I–II predominated in both men and women.

**Table 5:** Clinical outcomes at last follow-up.

Variables	All patients	Men	Women	*p*
	(*N* = 1,065)	(*n* = 546)	(*n* = 519)	
NYHA (*n* = 835; men *n* = 422, women *n* = 413)				0.401
1	365 (43.7)	194 (46.0)	171 (41.4)	
2	345 (41.3)	172 (40.8)	173 (41.9)	
3	112 (13.4)	51 (12.1)	61 (14.8)	
4	13 (1.6)	5 (1.2)	8 (1.9)	
Grade MR (*n* = 490; men *n* = 247, women *n* = 243)	55 (11.2)	30 (12.1)	25 (10.3)	0.515
Moderate—Severe	29 (5.9)	13 (5.3)	16 (6.6)	
Severe	26 (5.3)	17 (6.9)	9 (3.7)	
Grade AR (*n* = 844; men *n* = 439, women *n* = 405)	9 (1.1)	3 (0.7)	6 (1.5)	0.325
Moderate—Severe	8 (0.9)	3 (0.7)	5 (1.2)	
Severe	1 (0.1)	0 (0.0)	1 (0.2)	
Aortic mean gradient, mmHg **(*n*** **=** **449)**	8 (5.2–13.0)	8.2 (5.3–13.4)	8 (5.0–12.5)	0.456

NYHA, New York Heart Association functional class; MR, mitral regurgitation; AR, aortic regurgitation. Percentages use the number of patients with available data as the denominator. The peak-gradient field was excluded after data-quality review identified non-uniform historical coding.

Data are presented as *n* (%), mean ± standard deviation (SD), or median [interquartile range (IQR)].

Mitral regurgitation grade at latest follow-up was available in 490 patients (247 men and 243 women), and moderate/severe MR was uncommon and comparable between sexes. Aortic regurgitation grade was available in 844 patients (439 men and 405 women) and was likewise uncommon. Percentages in Table 5 use the number of patients with available data as the denominator.

Mean transprosthetic aortic gradient at latest follow-up was available in 449 patients and did not differ significantly between sexes (8.2 [5.3–13.4] mmHg in men vs. 8.0 [5.0–12.5] mmHg in women; *p* = 0.456). The historically coded peak-gradient field was excluded from the revised report after data-quality review identified non-uniform coding across procedural eras.

### 3.7 Multivariable analysis

The multivariable Cox analysis for 1-year mortality included 748 patients with complete covariate information and 86 deaths. Female sex was not independently associated with 1-year mortality (adjusted HR 1.07, 95% CI 0.69–1.66; *p* = 0.772). Higher baseline hemoglobin (adjusted HR 0.85, 95% CI 0.74–0.97; *p* = 0.015), higher eGFR (adjusted HR 0.99, 95% CI 0.98–1.00; *p* = 0.029), and higher LVEF (adjusted HR 0.97, 95% CI 0.95–0.99; *p* = 0.002) were associated with lower 1-year mortality. In a sensitivity analysis including procedure year as an additional continuous covariate, the association between sex and 1-year mortality remained non-significant. Complete primary Cox results are presented in Table 6.

**Table 6:** Multivariable cox proportional hazards regression analysis for 1-year all-cause mortality.

Variable	Adjusted HR	95% CI	*P*-value
Age, per year	0.99	0.95–1.03	0.717
Female sex	1.07	0.69–1.66	0.772
Frailty	1.10	0.68–1.78	0.685
Hemoglobin, per g/dL	0.85	0.74–0.97	0.015
eGFR, per mL/min/1.73 m²	0.99	0.98–1.00	0.029
Atrial fibrillation	0.88	0.55–1.40	0.594
STS score, per 1%	1.04	0.99–1.09	0.130
LVEF, per 1%	0.97	0.95–0.99	0.002
COPD	1.10	0.58–2.07	0.775

HR, hazard ratio; 95% CI, 95% confidence interval; eGFR, estimated glomerular filtration rate; LVEF, left ventricular ejection fraction; COPD, chronic obstructive pulmonary disease; STS, Society of Thoracic Surgeons. The multivariable model included 748 patients with complete covariate information and 86 deaths within 1 year. Missing covariate data were handled using complete-case analysis without imputation. The proportional hazards assumption was assessed using Schoenfeld residuals and was not violated globally (*p* = 0.226). Although eGFR showed evidence of non-proportionality, a sensitivity analysis incorporating a time-varying coefficient for eGFR yielded consistent results for female sex (adjusted HR 1.08, 95% CI 0.69–1.67; *p* = 0.747).

## 4 Discussion

### 4.1 Principal findings

Sex-related differences in cardiovascular diseases have been extensively described over recent decades, including differences in clinical presentation, diagnostic strategies, therapeutic approaches, and prognosis. In the context of TAVI, these differences are particularly relevant due to the distinct anatomical, physiological, and clinical characteristics observed between men and women. However, the available evidence remains heterogeneous, particularly in the contemporary TAVI era, which is marked by substantial technological advances and increasing operator experience (26).

In this context, the present study evaluated sex-related differences in a real-world cohort of patients undergoing TAVI between 2010 and 2023. The main findings were: 1) no significant differences in in-hospital, 30-day, and 1-year mortality; 2) a higher unadjusted incidence of bleeding in women, which was attenuated after multivariable adjustment, together with a non-significant trend toward increased vascular complications; and 3) a higher rate of new permanent pacemaker implantation in men that remained significant after adjustment.

#### 4.1.1 Sex and mortality after TAVI

The principal finding of our study was the absence of significant differences in in-hospital, 30-day, and 1-year mortality between men and women undergoing TAVI. Likewise, Kaplan–Meier and multivariable Cox analyses did not identify sex as an independent predictor of 1-year mortality.

These findings are consistent with contemporary evidence. See et al. reported no significant differences in 30-day or 1-year mortality between sexes after adjustment (HR 1.08, 95% CI 0.72–1.62; *p* = 0.72), suggesting that advances in device technology and procedural standardization have contributed to the attenuation of previously described sex-related disparities (27). Similarly, data from the Spanish TAVI Registry, including more than 12,000 patients, demonstrated comparable in-hospital, 30-day, and 1-year mortality between men and women, supporting the reproducibility of these findings in real-world practice (28).

Earlier studies suggested a potential survival advantage in women following TAVI (29). However, these analyses largely reflect earlier procedural eras characterized by first-generation devices and greater procedural heterogeneity. Subsequent improvements in preprocedural CT planning, reduction in delivery system profiles, and broader prosthesis sizing have likely contributed to improved procedural safety and more homogeneous outcomes across sexes. In addition, Deharo et al. reported that the apparent survival advantage in women was mainly driven by lower non-cardiovascular mortality, while cardiovascular mortality remained comparable between sexes (30).

Conversely, large administrative database analyses have reported slightly higher in-hospital mortality in women (31). Nevertheless, these differences appear to have progressively decreased over time, likely reflecting improvements in operator experience, patient selection, and device technology. Given that our cohort spans a long procedural period from 2010 to 2023, changes in operator experience, patient selection, and device technology may have influenced the observed outcomes and should be considered when interpreting sex-related comparisons.

A recent meta-analysis of 48 studies including 134,935 patients reported a time-dependent pattern, with women experiencing a long-term survival advantage after TAVI despite differences in early safety outcomes (38). Complementary risk-adjusted evidence after surgical aortic valve replacement has also suggested that early and late sex-related effects may differ, with female sex associated with better long-term survival despite less favorable early outcomes (40). These larger evidence bases indicate that small or time-dependent sex effects may not be detectable in individual single-center cohorts, particularly when follow-up is restricted to 1 year. Our neutral 1-year result should therefore not be interpreted as evidence that sex-related survival differences cannot emerge later.

Complementary evidence from a risk-adjusted meta-analysis of surgical aortic valve replacement similarly showed higher early mortality in women but better long-term survival, suggesting that sex-related effects after aortic valve replacement may be time-dependent and are not necessarily specific to TAVI. Differences in background life expectancy may also contribute to late survival patterns (40).

This issue is particularly relevant to sex-based analyses because adequately powered assessment of sex-related effects can require substantially larger samples than those needed for overall effects (39). Accordingly, the confidence interval around the sex estimate in our Cox model is compatible with both modest benefit and harm, and longer multicenter follow-up is required to assess late divergence in survival.

In our cohort, women were older, frailer, and had higher surgical risk scores, whereas men presented a higher burden of diabetes mellitus, chronic obstructive pulmonary disease, and renal dysfunction. These contrasting clinical profiles may have balanced overall mortality risk between groups. Accordingly, sex was not independently associated with mortality after adjustment for relevant baseline variables, suggesting that outcomes are primarily driven by comorbidity burden rather than biological sex *per se*.

In line with this, previous studies have identified renal dysfunction as a consistent predictor of mortality in both sexes, whereas other variables such as advanced NYHA class, atrial fibrillation, and baseline conduction abnormalities may exert sex-specific prognostic effects (27, 28).

Overall, our findings support the concept of the “sex paradox” in TAVI. Despite a higher burden of periprocedural complications in women, this does not translate into worse long-term survival. This may reflect underlying sex-related differences in cardiovascular pathophysiology, including more concentric remodeling and preserved left ventricular systolic function in women compared with more extensive calcific and dilated remodeling patterns in men, which is consistent with the higher baseline left ventricular ejection fraction observed in women in our cohort.

### 4.2 Bleeding and vascular complications

In our study, women had a higher unadjusted incidence of bleeding complications. However, the association between female sex and bleeding was attenuated after adjustment for age, body mass index, baseline hemoglobin, and eGFR, and no longer reached statistical significance. Vascular complications showed only a non-significant unadjusted trend toward a higher incidence in women.

Elbaz-Greener et al., using the Nationwide Inpatient Sample (NIS), reported significantly higher rates of bleeding (1.8% vs. 1.1%; *p* < 0.001) and vascular complications (5.1% vs. 3.7%; *p* < 0.001) in women, although a progressive decline in both outcomes was observed over time in both sexes. This improvement has been attributed to the widespread adoption of transfemoral access, lower-profile delivery systems, improved patient selection, and increased operator experience (32). Similarly, the meta-analysis by Lang et al., including more than 92,000 patients, demonstrated that male sex is associated with a substantially reduced risk of both vascular complications and bleeding events (33).

From a pathophysiological perspective, these differences are primarily explained by anatomical and morphometric factors. Supporting this, Ravani et al. reported a threefold higher incidence of life-threatening bleeding in women compared with men (6.1% vs. 2.4%; *p* < 0.01) in a contemporary registry (34). In addition, Kumar et al. demonstrated that these sex-related differences persist even after adjustment for baseline characteristics using propensity score matching, with women showing higher rates of vascular complications (1.2% vs. 0.7%; *p* < 0.001), post-procedural bleeding (2.34% vs. 1.72%; *p* < 0.001), and transfusion requirements (7% vs. 5.56%; *p* < 0.001) (31). These findings have been mainly attributed to smaller body surface area and more complex iliofemoral anatomy in women, which increase procedural complexity.

Finally, the meta-analysis of randomized controlled trials by Ferraz-Costa et al. confirmed a higher risk of major bleeding in women, supporting the existence of sex-related differences in periprocedural bleeding risk after TAVI (35). Importantly, these differences persist despite women typically presenting a lower prevalence of atherosclerotic risk factors such as diabetes mellitus and chronic kidney disease, as also observed in our cohort.

From a technical standpoint, smaller iliofemoral vessel dimensions in women can increase the sheath-to-femoral artery ratio and may contribute to vascular injury and bleeding. These observations support careful CT-based assessment of minimal luminal diameter, circumferential calcification and tortuosity, ultrasound-guided common femoral puncture when feasible, selection of the lowest-profile compatible delivery system, and meticulous closure-device planning. Because the present study did not directly compare access-planning strategies, these measures should be regarded as clinically plausible considerations rather than effects demonstrated by our data.

### 4.3 Permanent pacemaker implantation

Permanent pacemaker implantation remains one of the most frequent complications after TAVI and is associated with prolonged hospitalization and relevant long-term clinical implications. Among patients without a pre-existing permanent pacemaker, the rate of new permanent pacemaker implantation in our cohort was 22.5%, which is higher than that reported in a large meta-analysis including more than 70,000 patients (15.6%) (36), although it is consistent with contemporary European registries. In particular, the French TAVI Registry reported rates of 24.7% in men and 20.5% in women, whereas the Italian experience by Denegri et al. showed similarly elevated incidences of 26.1% and 21.2%, respectively (29, 30).

In our study, new permanent pacemaker implantation was more frequent in men than in women (26.9% vs. 17.8%). Importantly, this association persisted after adjustment for age, prosthesis category, and prosthesis size, with male sex associated with higher odds of new permanent pacemaker implantation. This finding is supported by multiple large-scale observational datasets. Amgai et al., using the Nationwide Inpatient Sample, reported a 15% lower likelihood of pacemaker implantation in women (37), and the Spanish TAVI Registry similarly confirmed higher rates in men (17.4% vs. 14.5%) (28).

Several mechanisms may explain this association. Men more frequently present baseline conduction abnormalities, particularly right bundle branch block, which increases susceptibility to post-procedural atrioventricular block. In addition, larger annular dimensions in men often necessitate implantation of larger prostheses, which may exert greater mechanical stress on the conduction system. By contrast, women typically receive smaller devices, potentially reducing direct interaction with the conduction tissue.

However, evidence is not entirely consistent across study designs. Recent randomized controlled trials and pooled analyses have not demonstrated significant sex-related differences in pacemaker implantation rates (35), suggesting that under more standardized procedural conditions, sex may lose its prognostic relevance. Similarly, See et al. reported comparable rates between sexes (11.4% in men vs. 11.2% in women), emphasizing the stronger influence of pre-existing conduction disease and procedural factors over biological sex *per se* (27).

Technological and procedural factors may modulate sex-related pacemaker differences. Prior work has suggested that prosthesis type can modify conduction risk; however, in our cohort the sex-by-prosthesis-category interaction was not significant (*p* = 0.953), providing no evidence that the sex association differed between self-expanding and balloon-expandable valves. The male association also remained similar after adjustment for procedure year. These findings should nevertheless be interpreted cautiously because baseline conduction variables and implantation-depth measurements were not consistently available.

### 4.4 Clinical implications

Our findings support individualized patient assessment before TAVI. Sex was not independently associated with mortality, but sex-related anatomical and clinical profiles may help contextualize procedural risk and inform, rather than determine, preprocedural planning.

Potential clinical implications include careful vascular-access assessment, particularly in women given their generally smaller peripheral vessel dimensions and the higher unadjusted bleeding rates observed in our cohort, and attention to established conduction-risk factors when assessing the risk of permanent pacemaker implantation. Although men had a higher adjusted likelihood of new pacemaker implantation, residual confounding by baseline conduction abnormalities and implantation-related factors cannot be excluded. These considerations require confirmation in datasets incorporating detailed CT anatomy, baseline conduction status, implantation depth, and contemporary device-specific information. Nursing care remains important for early complication recognition, patient education, and individualized follow-up, but the present observational study was not designed to test whether specific nursing or procedural strategies improve outcomes.

### 4.5 Study limitations

This study has several limitations. First, its retrospective, single-center design limits causal inference and generalizability, and residual confounding remains possible despite multivariable adjustment. In particular, the analysis of permanent pacemaker implantation could not account for potentially relevant conduction- and procedure-related factors, including baseline conduction abnormalities, implantation depth, and membranous septum, which were not consistently available. The sample size was substantial for a single-center cohort but was not specifically powered to detect small sex-related differences in infrequent outcomes or modest long-term survival effects.

Second, the inclusion period from 2010 to 2023 spans major changes in TAVI technology, CT planning, vascular-access techniques, patient selection, operator experience, and pacemaker-management practices. Procedure year was therefore examined in sensitivity analyses; adding procedure year did not materially change the estimated association of sex with bleeding, pacemaker implantation, or 1-year mortality. Nevertheless, year is only a proxy for multiple concurrent changes, and residual confounding by procedural era cannot be excluded.

Third, missingness varied across laboratory, echocardiographic, procedural, and follow-up variables. Analyses used available-case denominators for descriptive results and complete-case analysis for multivariable models without imputation. The Cox model therefore included 748 patients, and adjusted secondary-outcome models also used reduced complete-case samples. In addition, the institutional registry evolved over time and predominantly used VARC-2 event categories; independent event adjudication was not available. Finally, a historical peak-gradient field showed non-uniform coding and was removed from the revised report. These limitations reduce precision and may obscure small sex-related effects.

### 4.6 Future perspectives

Future research should focus on prospective, multicenter studies incorporating contemporary device generations, detailed procedural characteristics, and advanced imaging parameters to better elucidate the mechanisms underlying sex-related differences after TAVI.

Beyond traditional clinical endpoints, further investigation should explore sex-specific vulnerability patterns integrating functional status, frailty, and psychosocial factors. In particular, nursing-led research may contribute substantially to this field by evaluating the impact of structured follow-up programs, individualized patient education, and early detection strategies on outcomes such as quality of life, readmissions, medication adherence, and patient satisfaction.

Such approaches may support the development of more personalized care pathways and strengthen the integration of sex-specific considerations into cardiovascular nursing practice, ultimately improving equity and outcomes in patients undergoing TAVI.

## 5 Conclusions

In this real-world cohort, sex was not independently associated with 30-day or 1-year all-cause mortality after TAVI. Women experienced more bleeding in unadjusted analyses, although the association was attenuated after multivariable adjustment, whereas male sex remained associated with higher odds of new permanent pacemaker implantation. These associations should be interpreted as observational and hypothesis-generating and support further study of sex-related anatomical and procedural risk profiles rather than sex-based treatment decisions.

## Data Availability

The datasets presented in this article are not readily available because they contain potentially identifiable patient information and are subject to institutional and ethical restrictions. Requests to access the datasets should be directed to the corresponding author and will be considered on reasonable request, subject to approval by the corresponding institution. Requests to access the datasets should be directed to victor.alfonso.jimenez.diaz@sergas.es.
