# Supplementary material for: Sex differences in clinical outcomes after transcatheter aortic valve implantation: a real-world cohort
Source: Front Cardiovasc Med. 2026 Oct 20;13:1941651. doi: 10.3389/fcvm.2026.1941651 (PMC13727531; doi:10.3389/fcvm.2026.1941651)
Supplement: Supplementary file 1 [file Table1.docx]

**Table S1. Multivariable logistic regression analyses for any bleeding and new permanent pacemaker implantation.**

| **Variable** | **Adjusted OR** | **95% CI** | **P-value** |
| --- | --- | --- | --- |
| **Any bleeding (n=892; 57 events)** | | | |
| Female sex | 1.61 | 0.92–2.81 | 0.093 |
| Age, per year | 0.99 | 0.94–1.04 | 0.630 |
| Hemoglobin, per g/dL | 0.88 | 0.74–1.04 | 0.133 |
| eGFR, per mL/min/1.73 m² | 0.99 | 0.97–1.00 | 0.047 |
| BMI, per kg/m² | 1.00 | 0.94–1.05 | 0.899 |
| **New permanent pacemaker implantation (n=927; 208 events)** | | | |
| Male sex | 1.49 | 1.03–2.17 | 0.036 |
| Age, per year | 1.02 | 1.00–1.05 | 0.107 |
| Prosthesis category | 1.03 | 0.64–1.66 | 0.901 |
| Prosthesis size, per mm | 1.04 | 0.98–1.11 | 0.162 |

**Table S2. Sensitivity analyses.**

| **Outcome / analysis** | **Effect estimate** | **95% CI** | **P-value** |
| --- | --- | --- | --- |
| 30-day mortality: female sex* | OR 1.79 | 0.80–3.98 | 0.154 |
| Any bleeding: female sex, additionally adjusted for procedure year† | OR 1.57 | 0.90–2.75 | 0.114 |
| New permanent pacemaker: male sex, additionally adjusted for procedure year‡ | OR 1.48 | 1.02–2.16 | 0.040 |
| Sex × prosthesis category interaction for new pacemaker§ | OR 1.03 | 0.43–2.44 | 0.953 |
| 1-year mortality: female sex, sensitivity model including procedure year¶ | HR 0.92 | 0.59–1.45 | 0.720 |

* Logistic regression adjusted for age and STS score; date-based 30-day mortality population. † Bleeding model additionally included age, BMI, hemoglobin, eGFR, and procedure year. ‡ Pacemaker model additionally included age, prosthesis category, prosthesis size, and procedure year; patients with pre-existing pacemakers were excluded. § Interaction model included sex, prosthesis category, sex×prosthesis category, age, and prosthesis size. ¶ Procedure year was entered as a continuous covariate in a sensitivity Cox model; the primary model is reported in manuscript Table 6.
